# High Frequency of Macrolide-Resistant *Streptococcus pneumoniae* Colonization in Respiratory Tract of Healthy Children in Ardabil, Iran

**Published:** 2019-02

**Authors:** Khadije Mohammadi Gharibani, Ahad Azami, Masoomeh Parvizi, Farzad Khademi, Seyed Fazlullah Mousavi, Mohsen Arzanlou

**Affiliations:** 1 Department of Microbiology, Ahar Branch, Islamic Azad University, Ahar, Iran,; 2 Department of Internal Medicine, Imam Khomeini Hospital, Ardabil University of Medical Sciences, Ardabil, Iran,; 3 Department of Microbiology, School of Medicine, Ardabil University of Medical Sciences, Ardabil, Iran,; 4 Department of Microbiology, Pasteur Institute of Iran, Tehran, Iran.

**Keywords:** *S. pneumoniae*, Healthy children, Macrolide, Antibiotic resistance, *ermB*, *mefA/E*

## Abstract

**Background:**

*Streptococcus pneumoniae* (*S. pneumoniae*) is one of the most common causes of human diseases in young children. Macrolides are commonly antibiotics used for empirical treatment of community-acquired respiratory infections. The purpose of this study was to determine antibiotic resistance pattern as well as the relationship between macrolide resistance and the major mechanisms of resistance in pneumococci isolated from healthy children.

**Materials and Methods:**

In this cross-sectional study, 43 isolates of *S. pneumoniae* were collected from healthy children in Ardabil. Resistance pattern against tested antibiotics was determined using the disk diffusion method. The Minimum Inhibitory Concentration (MIC) of erythromycin was determined using the E-test strips. The *mefA/E* and *ermB* gene were detected in erythromycin-resistant isolates using the specific primers and Polymerase Chain Reaction (PCR) technique.

**Results:**

According to antimicrobial susceptibility testing, 74.4 % of the isolates were resistant to erythromycin, 95.3 % to penicillin, 81.3 % to co-trimoxazole, 72 % to azithromycin, 41.8 % to tetracycline, 27.9 % to clindamycin, and 16.2 % to chloramphenicol. All isolates were susceptible to levofloxacin and vancomycin. In the case of rifampin, 95.3% of the isolates were sensitive and 4.6% semi-sensitive. The MIC of erythromycin for resistant isolates was between 1.5 and ≥ 256 μg/ml. PCR results revealed that 100% of erythromycin-resistant isolates contained *mefA/E* gene and 81.25 % contained both the *ermB* and *mefA/E* genes.

**Conclusion:**

The prevalence of antibiotic-resistant strains of *S. pneumoniae*, especially resistance to macrolides, was high among healthy children in Ardabil. According to the results of this study, we suggest using levofloxacin, rifampin and vancomycin antibiotics as an appropriate prophylactic regimen in pneumococcal infections.

## INTRODUCTION

*Streptococcus pneumoniae* (*S. pneumoniae*) remains as one of the most important pathogens of human in the world (1). The bacterium is cause of life-threatening diseases such as sepsis, meningitis, pneumonia and otitis in children and immunocompromised elderly patients (2). Already, all isolates of *S. pneumoniae* were susceptible to penicillins; however, due to the emergence and spread of penicillin resistance, these antibiotics were replaced by other types, such as macrolides, lincosamides, streptogramin, ceftriaxone, cefotaxime and vancomycin (3–5). Macrolide antibiotics are group of broad-spectrum antibiotics containing erythromycin, azithromycin and clarithromycin which are used in order to treat respiratory infections. Erythromycin was the first macrolide discovered in 1952 and originally considered as an excellent alternative against penicillin-resistant gram-positive bacterial infections (6). However, failures in the treatment of pneumococcal infections with macrolide antibiotics have been reported earlier. High macrolide use is correlated with the increase of macrolide-resistant *S. pneumoniae* (7). Globally, macrolide resistance among *S. pneumoniae* is geographically variable but ranges from <10% to >90% of isolates (8). Local studies from Iran showed macrolide resistance ranges from 8.2–57.2% (9, 10) and >70% among *S. pneumoniae* isolates collected from healthy and sick subjects, respectively (11).

Understanding the antibiotic resistance pattern of *S. pneumoniae* is necessary for appropriate antibiotic treatment of pneumococcal infections. Therefore, this study was conducted to determine the extent of macrolide resistance and elucidate the major underlying mechanisms in *S. pneumoniae* isolates collected from healthy children less than six years old in Ardabil, Iran.

## MATERIALS AND METHODS

This cross-sectional study was conducted on 43 isolates of *S. pneumoniae* collected, using nasopharyngeal swab from 280 healthy children less than 6 years old attending kindergartens in Ardabil in 2015. The isolates were previously identified based on conventional methods and confirmed by presence of *lytA* gene using Polymerase Chain Reaction (PCR) method.

### 1. Antibiotic susceptibility testing

Antibiotic susceptibility testing was performed by disk diffusion method using Mueller-Hinton agar containing sheep blood (5%). The antibiotic disks were as follows: levofloxacin (LEV, 5 μg), trimethoprim-Sulfamethoxazole (SXT, 1.25/23.75 μg), clindamycin (DA, 2 μg), erythromycin (E, 15 μg), tetracycline (TE, 35 μg), chloramphenicol (C, 35 μg), azithromycin (AZM, 15 μg), penicillin (determined using oxacillin disk, 1 μg), vancomycin (VA, 35 μg), and rifampin (RA, 5 μg). The results were interpreted in accordance with the Clinical and Laboratory Standards Institute (CLSI) criteria (12).

The Minimum Inhibitory Concentrations (MICs) of erythromycin against isolates were determined using the E-test strips (Epsilon Test), with gradient concentrations ranging from 0.016 to 256 μg/ml. In this test, inhibition zone of growth was observed, pear shape, and the minimum concentration of antibiotic that inhibits the growth of bacteria is considered as the MIC value. According to the CLSI guideline, erythromycin susceptibility patterns are reported as follows: MIC ≤ 0.25 μg/ml as sensitive, 0.5 μg/ml as intermediate, and ≥ 1μg/ml as resistant.

### 2. Evaluation of inducible clindamycin resistance

Isolates that were sensitive to clindamycin and resistant to erythromycin tested for inducible resistance using the D-test. The test was performed by double-disk diffusion method. Erythromycin (15 μg) and clindamycin (2 μg) disks were placed close together within 20 mm apart from centre to centre on Mueller-Hinton agar plates. The plates were incubated overnight at 37 °C, D-shaped inhibition zone around the clindamycin disk adjacent to erythromycin disk indicated inducible clindamycin resistance (iMLSB). If an isolate was resistant to erythromycin but sensitive to clindamycin, without flattening of zone around clindamycin, was considered as MS phenotype. If the isolate was resistant to both erythromycin and clindamycin with circular shape of zone of inhibition was labeled as constitutive macrolidelincosamide-streptogramin B resistant phenotype (cMLSB) (12).

### 3. PCR amplification of **mefA/E** and **ermB** genes

Chromosomal DNA was extracted from erythromycin-resistant *S. pneumoniae* isolates using the DNPTMKit (CinnaGen, Iran) according to the manufacturer's protocol.

Quality and quantity of extracted DNA was assayed by measuring OD_260_ and OD_280_ nm using Nanodrop (Termo Scientific, USA) and then stored at −20 °C for subsequent uses. Specific primers were used to amplify the *mefA/E* (forward: 5′- AGT ATC ATT AAT CAC TAG TGC-3′ revers: 5′- TTC TTC TGG TAC TAA AAG TGG-3′) and *ermB* (forward: 5′- GAA AAG GTA CTC AAC CAA ATA-3′, revers: 5′- AGT AAC GGT ACT TAA ATT GTT TCA -3′) genes (13). PCR was performed in a 20 × μL AccuPower™ PCR PreMix (Bioneer) with 10 pmol of each primer under the following conditions: initial denaturation at 95°C for 5 min, followed by 34 cycles of 95°C for 1 min, 55°C (*ermB)* and 50°C (*mefA/E*) for 1 min and 72°C for 1 min, and a final incubation at 72°C for 5 min. The amplified DNA fragments (PCR products: *mefA/E*, 348 bp, and *ermB*, 639 bp) were separated on 1% (w/v) agarose gel, stained with ethidium bromide and visualized under ultraviolet light.

### Statistical analysis

Statistical analysis was carried out using SPSS software version 16.0. The associations of erythromycin resistance genotypes with resistance to other antibiotic classes were calculated using the chi-square test. Statistical significance was set at p< 0.05.

## RESULTS

In the present study, antibiotic susceptibility test was performed by disk diffusion method and the MIC through the E-test strips for each 43 pneumococcal isolates. As shown in table 1, according to the disk diffusion test 100% of the isolates were susceptible to vancomycin and rifampin and 95.40% for levofloxacin. Forty-one of 43 isolates (95.34%) were resistant to penicillin and for erythromycin 30 of 43 isolates (69.76%) were resistant, 2 (4.6%) were intermediate and 11 (25.5%) were susceptible. Based on the E-test, 32 isolates were resistant to erythromycin. In the present study, the most resistance was obtained to penicillin (95.34%), trimethoprim (81.3%), erythromycin (74.4%), azithromycin (72%) and tetracycline (41.86 %), respectively. Overall, 28% of isolates were resistant to clindamycin. No inducible resistance to clindamycin was observed.

**Table 1.** Antibiotic resistance patterns of *Streptococcus pneumoniae* strains isolated from children in Ardabil, Iran, using agar diffusion method

**Antibiotics**	**Total isolates (N = 43), n (%)**			**Genotypes**						

				***mef A/E* (N=32), n(%)**			***erm B + mef A/E* (N= 26), n(%)**			***P***
	**S**	**I**	**R**	**S**	**I**	**R**	**S**	**I**	**R**	
**Erythromycin**	11(25.6)	-	32(74.4)	-	-	32 (100)	-	-	26 (100)	1
**Azithromycin**	11(25.6)	1(2.3)	31(72.1)	3 (9.38)	-	29(90.62)	3 (11.53)	-	23(88.46)	0.8
**Clindamycin**	31(72.1)	-	12(27.9)	20 (62.5)	-	12(37.5)	15(57.70)	-	11(42.3)	0.65
**Tetracycline**	25(58.4)	-	18(41.86)	16 (50)	-	16(50)	11(42.30)	-	15(57.70)	0.44
**Levofloxacin**	43(100)	-	-		-	-	-	-	-	-
**Trimethoprim**	7(16.3)	1(2.3)	35(81.4)	5 (15.62)	-	27(84.37)	4(15.39)	-	22(84.61)	0.9
**Chloramphenicol**	36(83.7)	-	7(16.3)	27(84.37)	-	5(15.62)	22(84.61)	-	4(15.38)	0.8
**Penicillin^1^**	2(4.65)	-	41(95.34)	-	-	32(100)		-	26 (100)	1
**Vancomycin**	43(100)	-	-	-	-	-		-	-	-
**Rifampin**	41(95.34)	2(4.6)	-	-	-	-		-	-	-

S; Susceptible, I; Intermediate, R, Resistant

1 Determined using oxacillin disk, 1μg

As shown in table 2 the majority of the isolates were resistant against multiple classes of antibiotics. Overall, 74.60 % of isolates were resistant to ≥ 3 antibiotics classes tested.

**Table 2.** Antimicrobial susceptibility profile for *S. pneumoniae* isolates collected from children in Ardabil, Iran

Isolates N= 43 n (%)	Antibiotic resistance pattern	Antibiotic types n	Antibiotic class n	Total ^a^ n (%)
1(2.32)	-	0	0	1 (2.32)
1(2.32)	P	1	1	2 (4.65)
1(2.32)	SXT	1	1	
5(11.62)	SXT, P	2	2	8 (18.60)
1(2.32)	AZM, P	2	2	
2(4.65)	E, AZM, P	3	2	
1(2.32)	SXT, C, P	3	3	13 (30.20)
1(2.32)	DA, TE, P	3	3	
11(25.56)	SXT, E, AZM, P	4	3	
1(2.32)	SXT, E, C, P	4	4	7 (16.26)
1(2.32)	SXT, C, AZM, P	4	4	
1(2.32)	SXT, DA, TE, P	4	4	
4(9.30)	SXT, E, TE, AZM, P	5	4	
5 (11.61)	SXT, E, TE, C, AZM, P	6	5	12 (27.90)
3(6.97)	SXT, DA, E, TE, AZM, P	6	5	
4(9.30)	SXT, DA, E, TE, AZM, P	6	5	

a Total number of isolates resistant to same number of antibiotic class

Levofloxacin (LEV, 5μg), trimethoprim (SXT, 25μg), clindamycin (DA, 2μg), erythromycin (E, 15μg), tetracycline (TE, 35μg), chloramphenicol (C, 35μg), azithromycin (AZM, 15μg), penicillin (determined using oxacillin disk, 1μg), vancomycin (VA, 35μg), rifampin (RA, 5μg)

**Figure 1.**
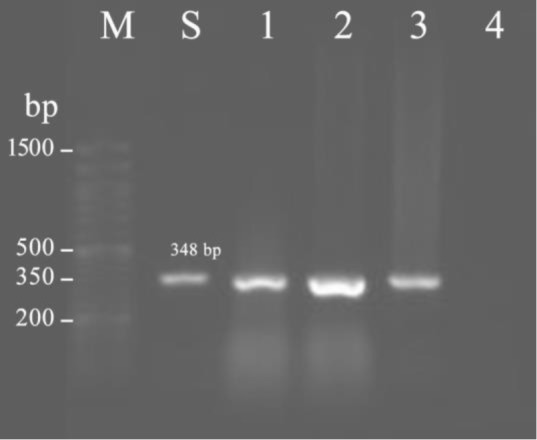
PCR detection of *mefA/E* gene in *Streptococcus pneumoniae* isolates. M: molecular weight markers, Lanes 1, 2 and 3 *mefA/E* positive isolates, naLe 4: Negative control, Lane S: Positive control.

**Figure 2.**
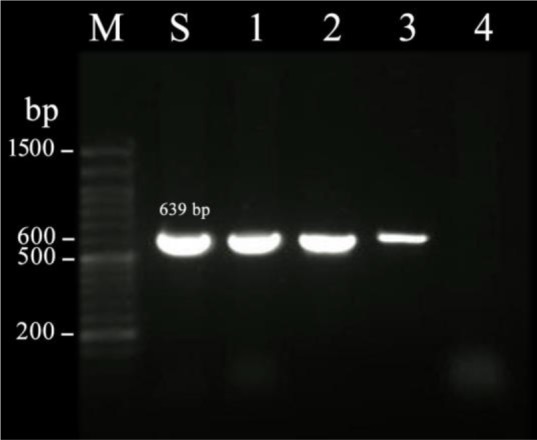
PCR detection of *ermB* gene in *Streptococcus pneumoniae* isolates. M: molecular weight markers, Lanes 1, 2 and 3 *ermB* positive isolates, Lane 4: Negative control, Lane S: Positive control strain PTCC 1240.

The MIC range for erythromycin was between 256 to ≥ 0.032 μg/ml and the MIC50 value was determined as 12 μg/ml. According to the MIC test results, 32 isolates of *S. pneumoniae* (74.4%) were resistant to erythromycin. The erythromycin MIC results in resistant isolates were variable between 1.5 and ≤256 μg/ml. The MIC50 value for erythromycin resistant isolates was 32 μg/ml. PCR testing revealed the presence of *mefA/E and ermB* genes in resistant isolates (Figures 1 and 2).

The *mefA/E* gene was detected in all of the erythromycin-resistant isolates and 26 (81.25%) of isolates had both the *mefA/E* and *ermB* genes. The erythromycin resistance level for isolates with both *mefA/E* and *ermB* genes was higher (MIC_50_= 48 μg/ml) in comparison with that of with *mefA/E* gene alone (MIC_50_= 32 μg/ml). There was no significant relationship between the erythromycin resistance genotypes and resistance to the antibiotics tested (p> 0.5) (Table 1).

## DISCUSSION

Macrolides are increasingly used in the treatment of diseases caused by *S. pneumoniae* (8). In this study, we evaluated the prevalence of macrolide resistance in *S. pneumoniae* isolates collected from healthy children in Ardabil. For erythromycin resistance, which is the most widely used macrolide drug, the results of the disk diffusion and E-test methods were not identical [30 (69.76%) vs. 32 (74.41%)]. This suggests that the E-test method is more accurate than disk diffusion method. Thirty-one (72.09%) isolates were resistant to azithromycin, a new semi-synthetic generation of macrolides, which is approximately identical to erythromycin. As compared to studies conducted in other cities of Iran, the prevalence of macrolide-resistant *S. pneumoniae* in healthy subjects in Ardabil was higher than in Kashan (8.2%), Zahedan (18.4%), Hamadan (25.5%), Mashhad (48.3%) and Tehran (57.2%) (9, 10, 14–16), as well as higher than the isolates collected from healthy children in Jordan, Hong Kong, Peru, Ghana, Uganda, Korea, Sri Lanka, Vietnam, Singapore, Thailand, China, India, Philippines and Russia (17–23). Previous studies showed a positive correlation between utilization of macrolides with the level of macrolide resistance in *S. pneumoniae* (24). Higher macrolide resistance in this study may be connected to the expanded utilization of macrolides in the study region. In a cross sectional study in 2016, it has been shown that antibiotics were contained within 54.9% of the prescriptions by general practitioners in Ardabil and macrolides were included in 18.3% of prescriptions (25).

Interestingly, the prevalence of erythromycin-resistant *S. pneumoniae* in healthy children in Ardabil was higher than children with pneumococcal infection in America (29%), Italy (3.4%), Finland (21.5%), Russia (19%), Greece (24%), Morocco (16.7 %), and Japan (4.69%) (26–31). While, it was lower than Vietnam (88.3%), Taiwan (87.2%), Korea (85.1%), Hong Kong (76.5%), and China (75.6%) (27). This finding is in accordance with previous reports showing that colonizer pneumococci isolates are more resistant as compared with invasive isolates (32). However, reports from Iran showed higher erythromycin resistance in clinical isolates. Macrolide resistance in clinical isolates of *S. pneumoniae* has been increasing steadily in the Iran. In 2001, 2011, 2016 and 2017, those erythromycin resistance rates were 25%, 65%, 75%, and 71.4%, respectively (11,33, 34).

Macrolide resistance in *S. pneumoniae* is mediated by three main mechanisms, including; (1) mutations in ribosomal proteins, (2) discharge of antibiotics due to efflux pumps and (3) changes in the structure of the target molecule through methylation of *23s rRNA* gene. The genes encoding efflux pumps *(mef A/E*) and methyltransferases enzymes (*erm B*) are carried on transposons, so spreading of resistance genes among the strains is possible (35).

In this study, we investigated erythromycin resistance genes by PCR method. Our results showed that the prevalence of *mefA/E* gene was higher than the *ermB* gene and 32 (100 %) of the erythromycin resistant isolates had *mefA/E* gene and 26 (81.25%) isolates had both *ermB* and *mefA/E* genes. Similar results were demonstrated in the agreement with this study in other countries. In Malaysia (64.7%), Hong Kong (66.7%) as well as Germany (50%) and Greece (*mefA* 5.3 % and *mefE* 41.8 %), the prevalence of *mefA* gene was more than *ermB* gene (30–39). However, frequency of *ermB* gene in some countries including Taiwan (70.7%), Sri Lanka (75%), China (76.9%) and Turkey (95%) were higher than *mefA* gene prevalence (30, 40). The study from other Iranian city has reported the similar finding as 42 and 50% for *ermB* and *mefA,* respectively (41). It has been documented that resistance mediated by the *ermB* gene, is usually associated with high-level macrolide MICs and efflux, encoded by the *mefA/E* gene, shows low-level macrolide MICs (30). Similar findings were observed in this study. MIC50 for isolates carrying both *ermB* gene was 48 μg/ml, whereas it was 32 μg/ml for isolates containing just *mefA/E* gene. However, in this study all isolates contained *mefA/E* gene and higher MICs in the presence of *ermB* gene could be at least partially attributed to the coexistence of *mefA/E* gene.

The results obtained from other antibiotics studied showed that 74.36 % of isolates were resistant to ≥ 3 antibiotic classes and had multiple drug resistance phenotypes. These results are inconsistent with recent reports from other regions (41). Most of the isolates were susceptible to levofloxacin, vancomycin, rifampin and chloramphenicol. These results were similar to the findings of other study conducted on clinical isolates collected in Iran (34).

In conclusion, because of the high resistance rate to macrolides, erythromycin and azithromycin, using these antibiotics is not recommended for empiric treatment of suspected pneumococcal infections in the study region. However, levofloxacin, rifampin and vancomycin can be used against infections caused by *S. pneumoniae* in Ardabil. Our study further demonstrated that *erm B* and *mef A/E* genes are dominantly present in macrolide resistant isolates. Due to the carrying of these genes by transposons, the isolates could act as reservoir for persistence and dissemination of macrolide resistant pneumococcal isolates in the community.

## References

[1] Engholm DH Kilian M Goodsell DS Andersen ES Kjærgaard RS A visual review of the human pathogen Streptococcus pneumoniae. FEMS Microbiol Rev 2017;41(6):854–879. 29029129 10.1093/femsre/fux037

[2] Obaro S Adegbola R The pneumococcus: carriage, disease and conjugate vaccines. J Med Microbiol 2002;51(2):98–104. 11863272 10.1099/0022-1317-51-2-98

[3] Livermore DM Bacterial resistance: origins, epidemiology, and impact. Clin Infect Dis 2003;36(Suppl 1):S11–23. 12516026 10.1086/344654

[4] Leclercq R Courvalin P Resistance to macrolides and related antibiotics in Streptococcus pneumoniae. Antimicrob Agents Chemother 2002;46(9):2727–34. 12183222 10.1128/AAC.46.9.2727-2734.2002 PMC127415

[5] Shi ZY Enright MC Wilkinson P Griffiths D Spratt BG Identification of three major clones of multiply antibiotic-resistant Streptococcus pneumoniae in Taiwanese hospitals by multilocus sequence typing. J Clin Microbiol 1998;36(12):3514–9. 9817864 10.1128/jcm.36.12.3514-3519.1998 PMC105231

[6] Zhanel GG Dueck M Hoban DJ Vercaigne LM Embil JM Gin AS Review of macrolides and ketolides: focus on respiratory tract infections. Drugs 2001;61(4):443–98. 11324679 10.2165/00003495-200161040-00003

[7] Skalet AH Cevallos V Ayele B Gebre T Zhou Z Jorgensen JH Antibiotic selection pressure and macrolide resistance in nasopharyngeal Streptococcus pneumoniae: a cluster-randomized clinical trial. PLoS Med 2010;7(12):e1000377.d 21179434 10.1371/journal.pmed.1000377 PMC3001893

[8] Schroeder MR Stephens DS Macrolide Resistance in Streptococcus pneumoniae. Front Cell Infect Microbiol 2016;6:98. eCollection 2016. 27709102 10.3389/fcimb.2016.00098 PMC5030221

[9] Mirzaei Ghazikalayeh H Moniri R Moosavi SG Rezaei M Yasini M Valipour M Serotyping, Antibiotic Susceptibility and Related Risk Factors Aspects of Nasopharyngeal Carriage of Streptococcus pneumoniae in Healthy School Students. Iran J Public Health 2014;43(9):1284–90. 26175983 PMC4500431

[10] Sanaei Dashti A Abdinia B Karimi A Nasopharyngeal carrier rate of Streptococcus pneumoniae in children: serotype distribution and antimicrobial resistance. Arch Iran Med 2012;15(8):500–3. 22827788

[11] Talebi M Azadegan A Sadeghi J Ahmadi A Ghanei M Katouli M Determination of Characteristics of Erythromycin Resistant Streptococcus pneumoniae with Preferred PCV Usage in Iran. PLoS One 2016;11(12):e0167803. 28033345 10.1371/journal.pone.0167803 PMC5199012

[12] CaLS I Performance standards for antimicrobial susceptibility testing: twenty-first informational supplement: CLSI document M100-S21. Wayne: Clinical and Laboratory Standards Institute 2011.

[13] Sutcliffe J Grebe T Tait-Kamradt A Wondrack L Detection of erythromycin-resistant determinants by PCR. Antimicrob Agents Chemother 1996;40(11):2562–6. 8913465 10.1128/aac.40.11.2562 PMC163576

[14] Bokaeian M Khazaei HA Javadimehr M Nasopharyngeal Carriage, Antibiotic Resistance and Serotype Distribution of Streptococcus Pneumoniae among Healthy Adolescents in Zahedan. Iran Red Crescent Med J 2011;13(5):328–33. 22737489 PMC3371970

[15] Mosleh MN Gharibi M Alikhani MY Saidijam M Vakhshiteh F Antimicrobial susceptibility and analysis of macrolide resistance genes in Streptococcus pneumoniae isolated in Hamadan. Iran J Basic Med Sci 2014;17(8):595–9. 25422753 PMC4240794

[16] Bakhshaee M Ghazvini K Naderi HR Zamanian A Haghighi J Boghrabadian M The prevalence of nasopharyngeal Streptococcal pneumonia carriers in Mashhad day care children and their antibiotic resistance pattern. Iran J Otorhinolaryngol 2006; 18(45):119–126.

[17] Al-Kayali R Khyami-Horani H van der Linden M Al-Lahham A Antibiotic resistance patterns and risk factors of Streptococcus pneumoniae carriage among healthy Jordanian children. Eur Int J Sci Technol 2016; 5: 55–76.

[18] Chiu SS Ho PL Chow FK Yuen KY Lau YL Nasopharyngeal carriage of antimicrobial-resistant Streptococcus pneumoniae among young children attending 79 kindergartens and day care centers in Hong Kong. Antimicrob Agents Chemother 2001;45(10):2765–70. 11557466 10.1128/AAC.45.10.2765-2770.2001 PMC90728

[19] Hanke CR Grijalva CG Chochua S Pletz MW Hornberg C Edwards KM Bacterial Density, Serotype Distribution and Antibiotic Resistance of Pneumococcal Strains from the Nasopharynx of Peruvian Children Before and After Pneumococcal Conjugate Vaccine 7. Pediatr Infect Dis J 2016;35(4):432–9. 26974749 10.1097/INF.0000000000001030 PMC4820239

[20] Dayie NT Arhin RE Newman MJ Dalsgaard A Bisgaard M Frimodt-Møller N Multidrug-Resistant Streptococcus pneumoniae Isolates from Healthy Ghanaian Preschool Children. Microb Drug Resist 2015;21(6):636–42. 26172078 10.1089/mdr.2014.0314

[21] Rutebemberwa E Mpeka B Pariyo G Peterson S Mworozi E Bwanga F High prevalence of antibiotic resistance in nasopharyngeal bacterial isolates from healthy children in rural Uganda: A cross-sectional study. Ups J Med Sci 2015;120(4):249–56. 26305429 10.3109/03009734.2015.1072606 PMC4816885

[22] Lee NY Song JH Kim S Peck KR Ahn KM Lee SI Carriage of antibiotic-resistant pneumococci among Asian children: a multinational surveillance by the Asian Network for Surveillance of Resistant Pathogens (ANSORP). Clin Infect Dis 2001;32(10):1463–9. 11317248 10.1086/320165

[23] Stratchounski LS Kretchikova OI Kozlov RS Reshedko GK Stetsiouk OU Tarasova GD Antimicrobial resistance of Streptococcus pneumoniae isolated from healthy children in day-care centers: results of a multicenter study in Russia. Pediatr Infect Dis J 2000;19(3):196–200. 10749458 10.1097/00006454-200003000-00004

[24] Bergman M Huikko S Huovinen P Paakkari P Seppälä H Finnish Study Group for Antimicrobial Resistance (FiRe Network) Macrolide and azithromycin use are linked to increased macrolide resistance in Streptococcus pneumoniae. Antimicrob Agents Chemother 2006;50(11):3646–50. 16940064 10.1128/AAC.00234-06 PMC1635217

[25] Hosseinzadeh F Sadeghieh Ahari S Mohammadian-erdi A Survey the antibiotics prescription by general practitioners for outpatients in Ardabil City in 2013. Journal of Ardabil University of Medical Sciences 2016;16(2):140–50.

[26] Felmingham D Grüneberg RN The Alexander Project 1996–1997: latest susceptibility data from this international study of bacterial pathogens from community-acquired lower respiratory tract infections. J Antimicrob Chemother 2000;45(2):191–203. 10660501 10.1093/jac/45.2.191

[27] Jacobs MR Felmingham D Appelbaum PC Grüneberg RN Alexander Project Group The Alexander Project 1998–2000: susceptibility of pathogens isolated from community-acquired respiratory tract infection to commonly used antimicrobial agents. J Antimicrob Chemother 2003;52(2):229–46. 12865398 10.1093/jac/dkg321

[28] Reinert RR Wild A Appelbaum P Lütticken R Cil MY Al-Lahham A Ribosomal mutations conferring resistance to macrolides in Streptococcus pneumoniae clinical strains isolated in Germany. Antimicrob Agents Chemother 2003;47(7):2319–22. 12821488 10.1128/AAC.47.7.2319-2322.2003 PMC161879

[29] de la Pedrosa EG Morosini MI van der Linden M Ruiz-Garbajosa P Galán JC Baquero F Polyclonal population structure of Streptococcus pneumoniae isolates in Spain carrying mef and mef plus erm (B). Antimicrobial agents and chemotherapy 2008;52(6):1964–9. 18362188 10.1128/AAC.01487-07 PMC2415790

[30] Song JH Chang HH Suh JY Ko KS Jung SI Oh WS Macrolide resistance and genotypic characterization of Streptococcus pneumoniae in Asian countries: a study of the Asian Network for Surveillance of Resistant Pathogens (ANSORP). J Antimicrob Chemother 2004;53(3):457–63. 14963068 10.1093/jac/dkh118

[31] Diawara I Zerouali K Katfy K Barguigua A Belabbes H Timinouni M Phenotypic and genotypic characterization of Streptococcus pneumoniae resistant to macrolide in Casablanca, Morocco. Infect Genet Evol 2016;40:200–204. 26961592 10.1016/j.meegid.2016.03.003

[32] García-Suárez Mdel M Villaverde R Caldevilla AF Méndez FJ Vázquez F Serotype distribution and antimicrobial resistance of invasive and non-invasive pneumococccal isolates in Asturias, Spain. Jpn J Infect Dis 2006;59(5):299–05. 17060695

[33] Haghi Ashtiani MT Sadeghian M Nikmanesh B Pourakbari B Mahmoudi S Mamishi S Antimicrobial susceptibility trends among Streptococcus pneumoniae over an 11-year period in an Iranian referral children Hospital. Iran J Microbiol 2014;6(6):382–6. 25926954 PMC4411422

[34] Houri H Tabatabaei SR Saee Y Fallah F Rahbar M Karimi A Distribution of capsular types and drug resistance patterns of invasive pediatric Streptococcus pneumoniae isolates in Teheran, Iran. Int J Infect Dis 2017;57:21–26. 28131730 10.1016/j.ijid.2017.01.020

[35] Charpentier E Tuomanen E Mechanisms of antibiotic resistance and tolerance in Streptococcus pneumoniae. Microbes Infect 2000;2(15):1855–64. 11165930 10.1016/s1286-4579(00)01345-9

[36] Sutcliffe J Resistance to macrolides mediated by efflux mechanisms. Curr Opin Anti-Infect Investig Drugs 1999;1: 403–12.

[37] Del Grosso M Camilli R Iannelli F Pozzi G Pantosti A The mef(E)-carrying genetic element (mega) of Streptococcus pneumoniae: insertion sites and association with other genetic elements. Antimicrob Agents Chemother 2006;50(10):3361–6. 17005818 10.1128/AAC.00277-06 PMC1610078

[38] Reinert RR Wild A Appelbaum P Lütticken R Cil MY Al-Lahham A Ribosomal mutations conferring resistance to macrolides in Streptococcus pneumoniae clinical strains isolated in Germany. Antimicrob Agents Chemother 2003;47(7):2319–22. 12821488 10.1128/AAC.47.7.2319-2322.2003 PMC161879

[39] Grivea IN Sourla A Ntokou E Chryssanthopoulou DC Tsantouli AG Syrogiannopoulos GA Macrolide resistance determinants among Streptococcus pneumoniae isolates from carriers in Central Greece. BMC Infect Dis 2012;12:255. 23057516 10.1186/1471-2334-12-255 PMC3484024

[40] Gulay Z Ozbek OA Bicmen M Gur D Macrolide resistance determinants in erythromycin-resistant Streptococcus pneumoniae in Turkey. Jpn J Infect Dis 2008;61(6):490–3. 19050364

[41] Raddaoui A Tanfous FB Chebbi Y Achour W Baaboura R Benhassen A High prevalence of multidrug-resistant international clones among macrolide-resistant Streptococcus pneumoniae isolates in immunocompromised patients in Tunisia. Int J Antimicrob Agents 2018;52(6):893–897. 29698665 10.1016/j.ijantimicag.2018.04.015

